# Lower Wages of Nurses in Long-Term Care: Does Racial and Ethnic Diversity Explain the Difference?

**DOI:** 10.1093/geroni/igab046.270

**Published:** 2021-12-17

**Authors:** Joanne Spetz, Laura Wagner, Timothy Bates

**Affiliations:** University of California, San Francisco, San Francisco, California, United States

## Abstract

Registered nurses (RNs) are a key component of the long-term care (LTC) workforce and prior research demonstrates their importance to ensuring patient safety in LTC settings. RNs who work in LTC settings earn less than those who work in hospitals and also are more likely to be from racial and ethnic minority groups. This study seeks to measure wage differences between Registered Nurses (RNs) working in LTC and other settings (e.g., hospitals) and whether differences are associated with the characteristics of the RN workforce between and within settings. We used the 2018 National Sample Survey of Registered Nurses (NSSRN) public-use file to examine RN employment and earnings. Our study population included a sample of 15,373 employed RNs who provided patient care. Characteristics such as race/ethnicity, type of RN degree completed, census region, and union status were included in bivariate analyses and multiple regression analyses to examine the effect of these characteristics on wages. Logistic regression was used to predict RN employment in LTC settings. We found that RNs in LTC experienced lower wages compared to those in non-LTC settings, yet this difference was not associated with racial/ethnic or international educational differences. LTC nurses were also significantly less likely to be represented by a labor union, and there was not a statistically significant wage difference for LTC RNs who were unionized. Because RNs in LTC earn lower wages than RNs in other settings, policies to minimize pay inequities are needed to support the RN workforce caring for frail older adults.

